# A False Alarm of Acute Abdomen: Epiploic Appendagitis Case Report and Literature Review

**DOI:** 10.7759/cureus.65529

**Published:** 2024-07-27

**Authors:** Noor ul Huda Ramzan, Talha Asif, Mahnoor Tauqeer, Muhammad Bilal Hashmat, Mian Uman Anwer

**Affiliations:** 1 Medicine, University Medical and Dental College, Faisalabad, PAK; 2 Internal Medicine, Allied Hospital, Faisalabad, PAK; 3 Internal Medicine, Allied Hospital, Faislabad, PAK

**Keywords:** fat attenuation, (nsaid) non-steroidal anti-inflammatory drugs, cost-effective practice, differential diagnoses of acute abdomen, primary epiploic appendagitis (pea)

## Abstract

An acute abdomen that is tender to palpation often represents a life-threatening emergency requiring immediate surgical or medical management. We present a case of acute abdomen with peritoneal signs and symptoms due to epiploic appendagitis (EA) that resolved with a single dose of ibuprofen. EA often mimics appendicitis, diverticulitis, and rarely cholecystitis based on its location. It arises due to ischemic infarction of an epiploic appendage, typically caused by torsion or spontaneous thrombosis of the central draining vein. Despite its rarity, clinicians need to recognize the characteristic imaging findings of EA on CT and ultrasound to avoid unnecessary surgical interventions and to manage the condition conservatively.

## Introduction

Epiploic appendagitis is a rare disease and has an incidence of approximately 8.8 cases per one million individuals annually [1]. The infarction of the epiploic appendage causes it and most commonly presents with acute abdominal pain in the left lower quadrant [2,3]. It is divided into two types, primary epiploic appendagitis (PEA) and secondary [4]. Primary epiploic appendagitis can occur due to inflammation resulting from torsion of the appendage, which is the most common cause, or due to occlusion of a vein secondary to thrombosis or an embolism [2]. Secondary epiploic appendagitis develops when inflammation extends from neighboring structures, such as in conditions like diverticulitis, appendicitis, or pancreatitis [4]. As it lacks specific clinical features, epiploic appendagitis is often mistakenly diagnosed as acute appendicitis or diverticulitis which can lead to overtreatment including hospitalization and surgery [2]. Due to the significant risks associated with misdiagnosing epiploic appendicitis, both in terms of patient health and financial implications, physicians must be mindful of this when diagnosing acute abdominal pain.

## Case presentation

The patient is a 44-year-old male with a history of atrial fibrillation on anticoagulation, who presented with sharp, epigastric pain for 24 hours. The pain started in the midline and then progressed to involve the left side without further radiation. He stated that the pain got worse when he sat up and reported difficulty in taking deep breaths due to the pain. He was not aware of any clear mitigating factors and also denied excessive physical activity or trauma. Lastly, he denied fevers, chills, nausea, vomiting, dysuria, hematuria, diarrhea, constipation, chest pain, and shortness of breath. He admitted to smoking marijuana daily and drinking up to three beers per day. On presentation, vitals were blood pressure of 133/74 mmHg, heart rate of 97 beats per minute, and respiratory rate of 18 breaths per minute with 98% saturation on room air. He was afebrile. An abdominal physical exam showed a soft abdomen that was profoundly tender to palpation in the epigastric and left lower quadrant with rebound tenderness and voluntary guarding. The positive peritoneal signs raised concerns for a potentially life-threatening intra-abdominal pathology, however, his lab work showed an unremarkable complete blood count, an unremarkable complete metabolic profile, and an unremarkable urinalysis. The rest of his lab work is outlined in Table 1 below.

**Table 1:** Lab work ordered for the patient All tests were within normal limits.

Lab study	Result	Reference range
Lactate level	0.9 mMol/L	0.5 - 2.2 mMol/L
Lipase level	37 Unit(s)/L	12 - 53 Unit(s)/L
High-sensitivity troponin	< 2 ng/L	Less than 45 ng/L

**Figure 1:**
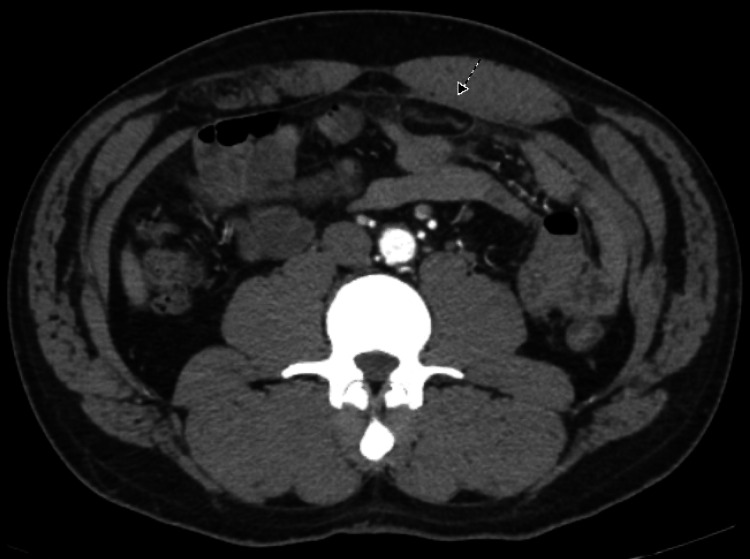
Computed tomography of the abdomen The arrow points to the 3.8 cm sharply circumscribed fat attenuation structure in axial view. It is surrounded by mild inflammation and is located in the anterior left mid-abdomen along the transverse colon.

**Figure 2:**
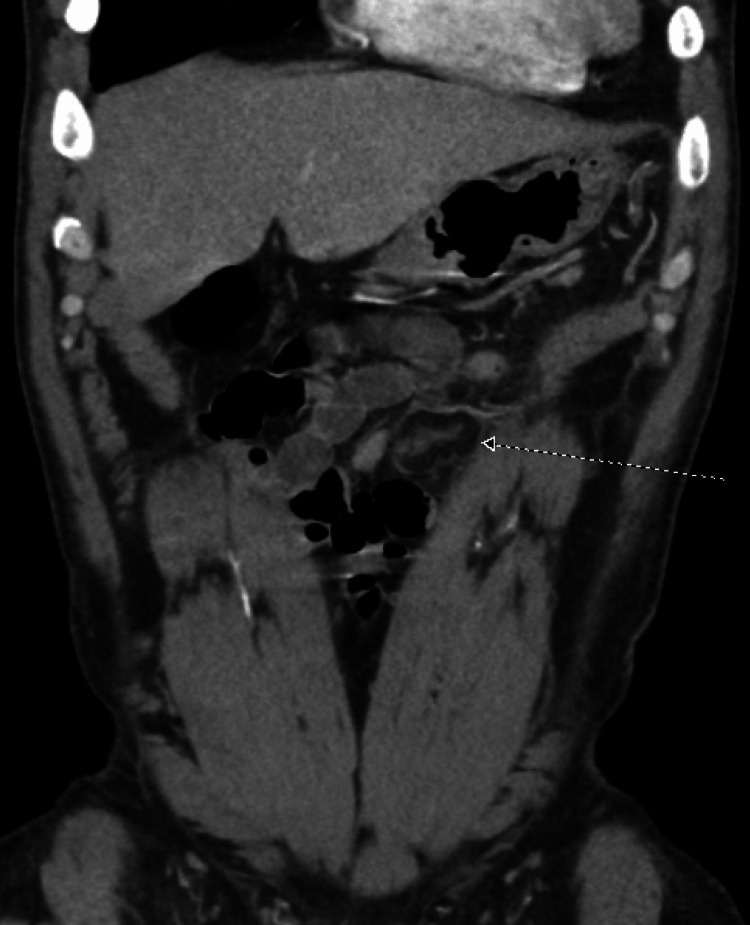
Computed tomography (CT) of the pelvis The arrow points to the 3.8 cm sharply circumscribed fat attenuation structure in coronal view. This is most consistent with epiploic appendagitis.

The cause of the severe abdominal pain remained unclear which prompted a computed tomography (CT) of the abdomen and pelvis. It showed a 3.8 cm sharply circumscribed fat attenuation structure with mild inflammation located in the anterior left mid-abdomen along the transverse colon, consistent with EA. This is shown in Figures 1, 2 below. The patient was given ibuprofen 600 mg with significant improvement in the pain. He was discharged with ibuprofen 600 mg every eight hours as needed but he denied using it at a follow-up appointment.

## Discussion

Epiploic appendages are small peritoneal outpouchings, ranging in size from 0.5 cm to 5 cm, and composed of adipose tissue arising from the outer wall of the large bowel [2]. They contain central vascular structures including branches of an end artery and a central vein. They occur in anterior and posterior rows along the tenia coli from the cecum to the rectosigmoid colon but are most frequently found in the transverse and sigmoid areas, usually in groups of 50 to 100 [2,5]. They have been hypothesized to work as a blood reservoir and assist in intestinal absorption and immunity but the exact function remains unknown [5].

EA is often associated with factors such as obesity, hernia, and exercise-related injuries and most commonly affects males aged thirty to fifty years [2]. Over a seven-year retrospective study, researchers observed that individuals with epiploic appendagitis had significantly higher abdominal adipose volume (60% more), visceral adipose area (117% more), and subcutaneous adipose area (35% more) than those in the control group [6]. It usually presents with acute abdominal pain in the lower part of the abdomen. An analysis of 21 patients found that 81% of the patients had pain in the left lower quadrant, 9.5% in the left middle, and 9.5% in the right lower quadrant [3]. The pain is usually non-radiating and may worsen with stretching of the abdomen and is rarely associated with nausea vomiting, diarrhea, constipation, loss of appetite, or other associated symptoms [5,7]. In a study by Choi et al., 25% of 31 individuals exhibited rebound tenderness, frequently resulting in misdiagnosis of conditions such as diverticulitis and appendicitis [7]. Unlike appendicitis and diverticulitis, epiploic appendagitis typically does not result in fever or elevated levels of white blood cells, erythrocyte sedimentation rate, or C-reactive protein [7,8]. Other differentials such as a biliary or a liver pathology can be ruled out with the absence of lab and vital sign abnormalities as well. 

As epiploic appendagitis is rare and lacks specific clinical features, it is frequently mistaken for more common medical conditions [9]. Accurately diagnosing this condition is important and a misdiagnosis can result in unnecessary antibiotic administration, hospitalization, and in some cases, surgery [2]. In the past, it was often discovered incidentally during surgeries intended to address other causes of acute abdominal pain [5]. In today’s age, it can be diagnosed with considerable accuracy using a CT scan [8]. It appears as an oval-shaped lesion measuring approximately 1-5 cm, surrounded by a region of increased density due to inflammation of the visceral peritoneum [8]. This hyperattenuating ring is highly suggestive of epiploic appendagitis and is used as a criterion for diagnosis [8]. Abdominal ultrasound can also diagnose the condition but may miss the diagnosis in some cases [8].

Epiploic appendagitis is a benign condition that typically self-resolves and can be treated conservatively with non-steroidal anti-inflammatory drugs (NSAIDs) for symptom relief [2,5,9,10]. Most patients recover within one to four weeks with conservative management [9]. Due to a lack of awareness about the condition, antibiotics have been widely administered to patients with epiploic appendagitis. A study of 31 patients with the condition found that 30 had received antibiotics [7]. However, there is no proven therapeutic advantage to antibiotic use [10]. It is also important to advise patients to seek medical attention if their condition worsens as it can signal the onset of complications such as obstruction, abscess formation, or peritonitis. Surgical intervention and antibiotics may be necessary for management in such cases [5,9].

## Conclusions

Our case falls in the most common epidemiologic group of males aged 30 to 50 years. The location of pain was also in the most commonly referenced location, the left side of the abdomen. Notably, there was a worsening of the pain with movement and significant rebound tenderness. Lastly, a CT scan of the abdomen showed a 3.8 cm highly attenuated ring in the region of the transverse colon leading to the diagnosis of epiploic appendagitis. Keeping in line with the literature and previous case studies, we opted to treat our patient conservatively and trial the use of NSAIDs. The patient experienced significant improvement in his symptoms and aggressive interventions were avoided. 

We recommend clinicians keep this rare condition on their differential when evaluating patients presenting with acute abdomen. However, further research is still needed to enhance our understanding of this condition and raise awareness among medical professionals.

## References

[1] de Brito P, Gomez MA, Besson M, Scotto B, Huten N, Alison D (2008). Frequency and epidemiology of primary epiploic appendagitis on CT in adults with abdominal pain (Article in French). J Radiol.

[2] Suresh Kumar VC, Mani KK, Alwakkaa H, Shina J (2019). Epiploic appendagitis: an often misdiagnosed cause of acute abdomen. Case Rep Gastroenterol.

[3] Chen JH, Wu CC, Wu PH (2011). Epiploic appendagitis: an uncommon and easily misdiagnosed disease. J Dig Dis.

[4] Mert A, Mırcık E (2021). Primary epiploic appendagitis: a case report. Cureus.

[5] Patel H, Abdelbaki A, Steenbergen P, Chanana C, Li S (2018). Know the name: acute epiploic appendagitis-CT findings and review of literature. AME Case Rep.

[6] Nugent JP, Ouellette HA, O'Leary DP, Khosa F, Nicolaou S, McLaughlin PD (2018). Epiploic appendagitis: 7-year experience and relationship with visceral obesity. Abdom Radiol (NY).

[7] Choi YU, Choi PW, Park YH (2011). Clinical characteristics of primary epiploic appendagitis. J Korean Soc Coloproctol.

[8] Giambelluca D, Cannella R, Caruana G (2019). CT imaging findings of epiploic appendagitis: an unusual cause of abdominal pain. Insights Imaging.

[9] Yang L, Jia M, Han P (2019). Primary epiploic appendagitis as an unusual cause of acute abdominal pain in a middle-aged male: A case report. Medicine (Baltimore).

[10] Giannis D, Matenoglou E, Sidiropoulou MS, Papalampros A, Schmitz R, Felekouras E, Moris D (2019). Epiploic appendagitis: pathogenesis, clinical findings and imaging clues of a misdiagnosed mimicker. Ann Transl Med.

